# An outbreak caused by GII.17 norovirus with a wide spectrum of HBGA-associated susceptibility

**DOI:** 10.1038/srep17687

**Published:** 2015-12-07

**Authors:** Xu-Fu Zhang, Qiong Huang, Yan Long, Xi Jiang, Ting Zhang, Ming Tan, Qiao-Li Zhang, Zhen-Yu Huang, Yue-Huan Li, Yao-Quan Ding, Gui-Fang Hu, Shixing Tang, Ying-Chun Dai

**Affiliations:** 1 School of Traditional Chinese Medicine, Southern Medical University, Guangzhou, Guangdong, China; 2 Department of Epidemiology, Guangdong Provincial Key Laboratory of Tropical Disease Research, School of Public Health and Tropical Medicine, Southern Medical University, Guangzhou, Guangdong, China; 3 Guangdong Provincial Center for Disease Control and Prevention, Guangzhou, China; 4 Divisions of Infectious Diseases, Cincinnati Children’s Hospital Medical Center; 5 Department of Pediatrics, University of Cincinnati College of Medicine, Cincinnati, Ohio, USA; 6 DongGuan Center for Disease Control and Prevention, Dongguan, Guangdong, China

## Abstract

During the past norovirus (NoV) epidemic season, a new GII.17 variant emerged as a predominant NoV strain, surpassed the GII.4 NoVs, causing outbreaks of acute gastroenteritis (AGE) in China. Here we report a study of an AGE outbreak in an elementary school in December 2014 caused by the new GII.17 NoV to explore the potential mechanism behind the sudden epidemics of the GII.17 NoV. A total of 276 individuals were sick with typical NoV infection symptoms of vomiting (93.4%), abdominal pain (90.4%), nausea (60.0%), and diarrhea (10.4%) at an attack rate of 5.7–16.9%. Genotyping of the symptomatic patients showed that individuals with a secretor positive status, including those with A, B, and O secretors and Lewis positive blood types, were sensitive to the virus, while the non-secretors and the Lewis negative individual were not. Accordingly, the recombinant capsid P protein of the GII.17 isolate showed a wide binding spectrum to saliva samples of all A, B, and O secretors. Thus, the broad binding spectrum of the new GII.17 variant could explain its widely spread nature in China and surrounding areas in the past two years.

Noroviruses (NoVs) are the leading cause of non-bacterial acute gastroenteritis (AGE) in both developed and developing countries and are responsible for over 50% of all acute gastroenteritis outbreaks worldwide^1^,^2^. NoVs consist of six genogroups (GI to GVI)^3^, among which GI and GII that contain 9 and 22 genotypes^4^, respectively, are responsible for most human infections. Over the past two decades, new variants of GII.4 have emerged approximately every 2–3 years, and were estimated to be responsible for 55–85% of NoV-associated outbreaks worldwide^2^. It was noted that during 2014–15 NoV epidemic season, a new GII.17 variant emerged locally in Guangdong and Jiangsu Province of China and Japan^5^,^6^,^7^. This newly emerged GII.17 variant surpassed GII.4 and became the dominant strain in causing NoV outbreaks in those Provinces of China^5^,^8^.

NoVs recognize human histo-blood group antigens (HBGAs) as attachment factors^9^. The association between the susceptibility of NoV infection and the host HBGA phenotypes has been established by human challenge studies and outbreak investigations^10^,^11^. The potential target populations and prevalence of individual genotypes of human NoVs fit well with their HBGA-binding profiles and the distributions of different ABO, secretor and Lewis types in the target populations^12^,^13^. Therefore, it is of significance to define the host susceptibility to GII.17 NoV infection in association with HBGA phenotypes. Here, we report our study on an AGE outbreak in a primary school caused by the newly emerged GII.17 variant.

Our data showed that this new GII.17 variant can infect all A, B and O secretors and accordingly, the recombinant P domain protein of the variant bound saliva of these individuals. Thus our data established the association between the HBGA phenotypes and infection of this new GII.17 variant.

## Results

### An AGE outbreak caused by a single GII.17 NoV

**Figure 1:**
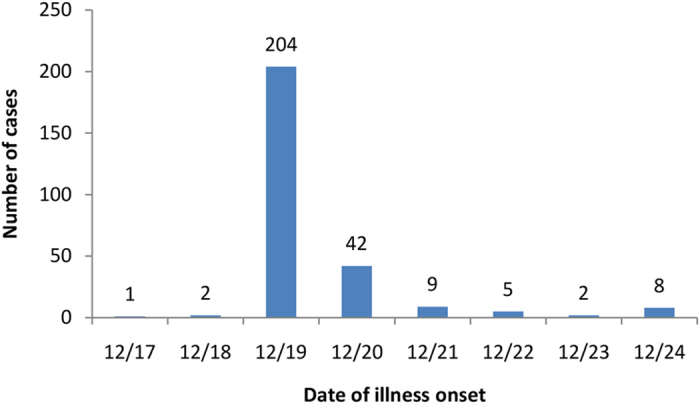
Epidemic curve of illness (n = 260) among students during the outbreak.

The outbreak occurred in a primary school with 2355 students and 80 teachers. One canteen with seven food workers provided breakfast for about 600 students and lunch for about 800 students. A total of 273 students, two food workers and one teacher were sick with AGE that met the NoV outbreak disease criteria. The first symptom onset (the index case) started on December 17^th^, while final case occurred on December 24^th^ of 2014 (Fig. 1). The median age of cases was 7 years (range 6–50 years), the majority of the cases (98.9%) were students. Attack rates among student respondents were 16.9% (first grade), 11.6% (second grade), 12.2% (third grade), 10.2% (fourth grade), 6.5% (fifth grade), and 5.7% (sixth grade) with significant decrease by grade (Χ^2^ = 34.8, df = 5, P < 0.001), in consistent with decreasing of the fraction of students per school-year eating lunch and breakfast in the canteen.

The questionnaires were obtained from 260 students, in which 115 (44.2%) were female, 169 (65.0%) had breakfast and 211(81.1%) had lunch in the school. Symptoms among the 260 students included vomiting (93.4%), abdominal pain (90.4%), nausea (60.0%), abdominal distension (17.3%), dizziness (12.7%), diarrhea (10.4%), fever (7.7%) and headache (6.5%). 107 (41.2%) of the 260 students sought medical attention in outpatient clinics or hospitals. Analysis of food exposures among the 260 students did not identify any food that showed statistically significant association with the illness during the three days before the index case.

Stool swab and vomitus samples were collected from 18 students, two food workers, one teacher with AGE on December 22^nd^ for laboratory tests. GII NoVs were detected in 14 (66.7%) of the 21 specimens by real-time RT-PCR, among which 13 samples were sequenced and identified as GII.17 NoVs (Table 1). All the specimens were also tested for Staphylococcus aureus and Bacillus cereus but neither bacteria were found. The 13 GII.17 NoV sequences showed 97–100% nucleotide identity, belonging to the same genetic cluster of the recently emerged GII.17 variant found in the Guangdong province^7^ (data not shown). Thus the studied AGE outbreak was caused by the single GII.17 variant.

### HBGA phenotypes and host susceptibility to symptomatic infection

**Figure 2:**
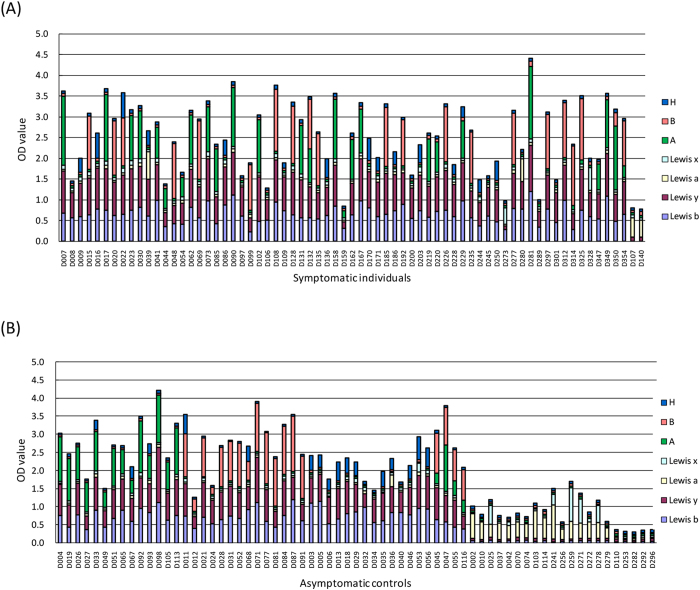
Profile of ABH and lewis antigen types in saliva samples. For symptomatic individuals group (**A**), all 69 samples were shown. For asymptomatic controls group (**B**), the saliva samples shown were composed of randomly selected 46 secretors and all 21 nonsecretors.

Saliva samples from 69 symptomatic individuals and 123 asymptomatic controls were tested for the ABO, Lewis, and secretor phenotypes. Typical distribution of ABO bloodtypes was seen among the asymptomatic controls and comparable with the general Chinese population (http://www.bloodbook.com/world-abo.html) (Table 2). In the present study, individuals with Le^a−b+^ and/or Le^x−y+^ and those with Le^a+b+^ or Le^x+y+^ phenotypes were grouped into secretors. In contrast, individuals with Le^a+b−^ and/or Le^x+y−^, Le^a−b−x−y−^ but lacking A, B, H phenotypes were sorted into non-secretors^14^. As a result, 67 secretors and two nonsecretors were found among symptomatic individuals; while 102 secretors and 21 nonsecretors were found among asymptomatic controls. Except the Lewis negative non-secretor group (n = 5) that were found only in asymptomatic controls, all other investigated HBGA phenotypes were found in the symptomatic individuals (Fig. 2 and Table 2).

Compared with controls, a significantly higher proportion of secretors and lower proportion of nonsecretors were found in the symptomatic group than the asymptomatic group (P = 0.003). No significant differences were seen in the proportions of blood types between symptomatic vs. asymptomatic individuals (P = 0.340). These data indicated that the secretor status correlated with infection of the GII.17 variant, the cause of this outbreak.

Similar scenario were seen, when the odds ratios were analyzed. Secretor individuals were at significantly higher infection risk than nonsecretors (OR = 6.90, 95% CI 1.57–30.38 vs. OR = 0.15, 95% CI 0.03–0.64, P = 0.003), including those with Le^a+b+^ or Le^x+y+^ (OR = 3.60, 95% CI 1.32–9.84) and without Le^a+b+^ or Le^x+y+^ (OR = 0.20, 95% CI 0.04–0.90) (Table 3). For symptomatic infection risk, no significant difference was found among individuals with different secretor bloodtypes (Table 3). In addition, no correlations were found between blood types and secretor status with clinical symptoms (Table 4).

### The HBGA Binding profile of the GII.17 variant

**Figure 3:**
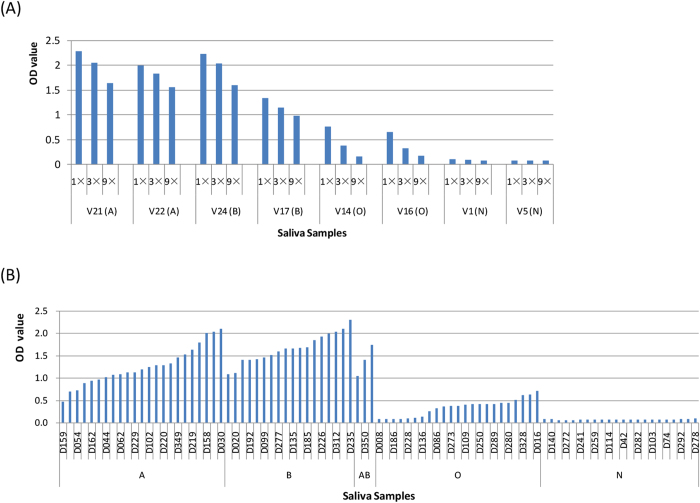
Saliva-based HBGA binding assay of P particle of GII.17 strain DG42. (**A**) 8 boiled well-characterized saliva samples were coated onto 96-well plates prior to the addition P particle. The P particle was tested in a series of 3-fold dilutions (15 μg/ml, 5 μg/ml and 1.7 μg/ml) by enzyme-linked immunosorbent assay (ELISA). (**B**) The binding activities of 90 saliva samples (all 69 symptomatic and 21 asymptomatic nonsecretor individuals) from the outbreak were sorted by blood types. The P particle was diluted at 5 μg/ml. “A,” “B,” “O,” and “N” represent the type A, B, O and nonsecretor saliva, respectively.

To further define the HBGA binding pattern and affinity of the GII.17 variant, P particles of this variant (DG42) were expressed and tested through the saliva-based HBGA binding assay. The results showed that the P particles of the new GII.17 variants bound saliva of type A, B, and O secretors in a dose-dependent manner (Fig. 3A), but not to saliva of nonsecretors. The saliva of secretors from the outbreak also bound the P particles (Fig. 3B), consistent with observed host HBGA susceptibility. The binding signals of the variant to saliva of type O secretors were weaker than those of type A, B or AB individuals (Fig. 3B), which was similar to GII.4 NoV binding pattern^15^.

### The HBGA-binding domain of the new GII.17 variant

**Figure 4:**
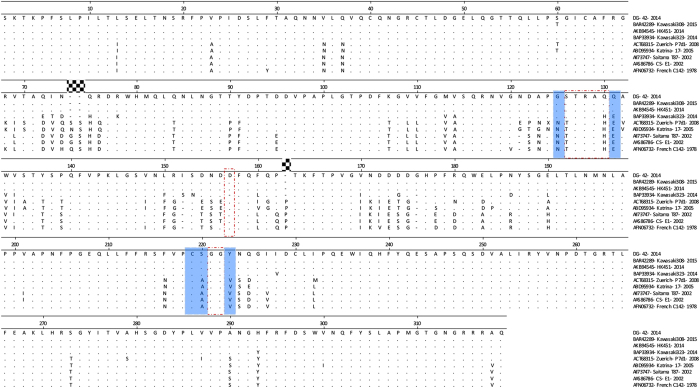
Comparison of P domain amino acid of the GII.17 outbreak virus (DG42) with other GII.17 strains. GII.17 strains were composed of those active in neighboring regions and countries during 2014-15 NoV epidemic season (Genbank accession number: BAR42289, AKB94545 and BAP33934) and strains circulating in the past decades (Genbank accession number: ACT 368315, ABD95934, AII73747, AAS86786 and AFN06732). The three conserved HBGA-binding interfaces are indicated by red empty rectangles (dashed lines), the surrounding amino acids of HBGA binding site are marked by blue shadow. Residues in the binding site and surrounding regions were highly conserved during 1978–2008. Two mutations in the conventional GII HBGA binding sites and four residues in the surrounding regions next to the binding site were also different for the newly emerged variant of 2014-15 NoV epidemic seasons.

**Figure 5:**
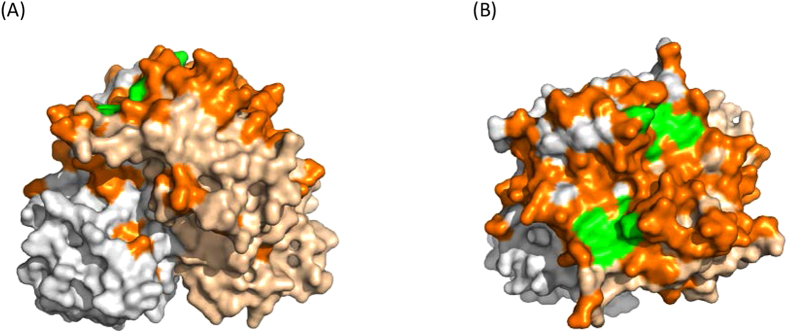
Indication of binding site and mutation in or near the binding site on GII.17 P domain homology model in side (A) and top (B) view. P monomer was in gray, binding pocket with green and mutation in or near the binding site in orange. The model was used to show the binding pocket and locations of the mutations of the GII.17 variant DG42 compared with the CS-E1 (2002) and the Kawasaki 323 (2014) variants are shown. Gray, P monomer; green, binding pocket; orange, mutation in or near the binding site.

To understand the possible reason for the suddenly increased prevalence of GII.17 NoVs^7^,^16^, we analyzed the changes of HBGA binding sites of the major GII.17 variants circulated in the past 37 years (1978–2015) focusing on the outbreak strain DG42 .We noted that the DG42 P domain share 93.1–100% amino acid identity with the variants causing epidemics during 2014-15 season, but only 78.7–80.6% homology with previous GII.17 variant that circulated during the past three decades. It was also noted that residues constituting the conserved HBGA binding site and its surrounding regions remained highly conserved during the past 35 years (1978–2013), until the newly emerged variant appeared in 2014 (Fig. 4). Two major mutations occurred in the GII.17 HBGA binding site^17^ of the newly emerged variant compared with the earlier strains. In addition, four residue mutations in the surrounding regions of the binding site were also seen (Figs 4 and 5).

## Discussion

As a rarely reported genotype, GII.17 remained silent for the past decades^18^,^19^. However, in the past NoV epidemic season, several most recent report showed that a newly emerged GII.17 variant caused major outbreaks in China and Japan, making it a dominant strain surpassing GII.4 in these regions^5^,^6^,^7^,^8^. This sudden increased prevalence of GII.17 may be caused by changes of certain virus-related factor(s) that remain elusive. For example, changes at the antigenic epitopes of the viral capsid that may lead to new adaptive advantage for rapid spread^5^,^6^. In addition, changes at the HBGA binding site and/or its surrounding region may result in host range expansion. To explore such change as possible mechanism to explain the increased prevalence of the new GII.17 variant, we investigated an AGE outbreak caused by the same GII.17 variant, focusing on the host susceptibility in association with the HBGA phenotypes. Our data showed that the infection source of the outbreak could be the chief cook (food worker 1) who was infected and shedded NoVs at 3 days before the outbreak. Another food worker might be infected from him. Students were the major implicated population.

Previous studies have shown a strong association between HBGA phenotypes and NoV infection, which is believed to be an important factor affecting the prevalence of GII NoVs^20^,^21^. For example, the GII.4 NoVs recognize HBGAs of all A, B, O secretors^22^ that represent over 80% of the general population, and some strains can even recognize nonsecretor^13^,^14^, which explains their predominance in causing NoV epidemics worldwide. Further studies showed that GII.4 NoV P domains can accommodate numerous HBGA types^23^,^24^. In the present study, we found that the newly emerged GII.17 variant can also infect individuals with all A, B and O secretors, including a low rate of non-secretors, suggesting that this GII.17 variant also has a wide spectrum of target populations. This finding not only explains the emerging epidemic caused by this new GII.17 variant in China, but also alert the global surveillance system for a potential pandemic of this newly emerged GII.17 variant in the near future^16^.

This new GII.17 variant was also detected in neighboring regions and countries of Asia, including Japan^7^, Hong Kong, Taiwan^25^, and United States^26^. GII.17 NoV was also found in groundwater in Kenya^27^, suggesting that this variant of GII.17 has been active in these areas in recent years. Compared with other GII.17 variants that circulated previously, the new variant found in this study had significant sequence variations and was classified into cluster C^5^,^7^,^26^. Two amino acid mutations were noted in the HBGA binding site, while four other mutations were seen in the surrounding regions of the binding site (Figs 4 and 5). As indicated by previous studies^17^,^28^,^29^,^30^, these mutations may result most likely in changes of HBGA binding property of the new GII.17 variant, which in turn, may expand its host range and become more prevalent^31^.

One limitation of our study is that some controls may be asymptomatically infected or have not been exposed to the virus in this case-control study. However, it is unlikely to influence the main finding that GII.17 can infect individuals with a wide spectrum of different HBGA phenotypes. Continued monitoring of emerging GII.17 strains is highly demanded for a better understanding of their evolution and epidemiology.

## Methods

### Outbreak investigation and sample collection

In December 2014, a NoV gastroenteritis outbreak occurred at a primary school (December 17–24) in DongGuan city, GuangDong Province, China. Illnesses were reported among students, food workers, and teacher. Epidemiologic investigations indicated that one chief cook (food worker 1, Table 1) was ill with 8–10 episodes diarrhea per day since December 14^th^ 10 pm, suggesting the cook as the probable source of NoV contamination in the food.

Description of symptoms and exposure history were obtained through a questionnaire sent to all students with symptoms in the study. Cases with NoV infection and disease were defined by at least one of the following signs or symptoms^11^: vomiting, diarrhea, or nausea combined with stomach cramp after December 17^th^. 21 samples from individuals with NoV diseases were collected, including 14 stool swab and 4 vomitus samples from students, 1 stool swab sample from teacher and 2 stool swab samples from food workers.

To test the role of HBGAs in the host susceptibility to the outbreak strain, saliva samples were collected using multiple-stage sampling, as follow: 1) symptomatic students distributed in 28 classes out of totally 42 classes, among which 12 classes with more than 10 cases per class. To improve the efficiency of this study, these 12 classes were selected in the first stage. 2) 6 out of 12 classes with more than 10 cases per class were randomly selected and 339 saliva samples were collected from all students in these 6 classes at second stage. 3) All 69 saliva samples from symptomatic students and almost half systematic sampling saliva samples (123 out of 270) from asymptomatic students were used to test for HBGA phenotyping at third stage. Together, 192 students in the 6 classes were included in this study. Stool and saliva samples collection was approved by the ethics committee of Guangdong Center for Disease Control and Prevention (GDCDC-W96-027B-2014.100). Informed consent was obtained by parents or child. All experiments were performed in accordance with relevant guidelines and regulations.

### NoV detection, cloning and sequencing of the P domain

Stool specimens were tested for Staphylococcus aureus and Bacillus cereus by culture. NoV was firstly tested by one-step real-time reverse transcription-polymerase chain reaction assay (RT-PCR, using GI and GII primers) in DongGuan Center for Disease Control and Prevention^32^. To further genotyping, One-Step RT-PCR (QIAGEN, CA, USA) was performed with region C-specific primers^33^. The positive PCR products were sequenced and genetic identity of the viruses was determined using the NoV Automated Genotyping Tool^34^.

The P domain sequences of positive samples were amplified using primers based on sequences of a previously reported GII.17 strain Kawasaki 323 (AB983218), and then cloned into T-vector (Thermo Fisher, US) before being sequenced (Invitrogen, CN). Alignment of P domains was performed using MegAlign of DNAstar 7.0. HBGAs binding residues of GII.17 viruses were determined by the high conservation of the residues constituting the CBPs (central binding pockets) in sequence alignments of NoV P domains^12^,^35^,^36^.

### Preparation of P particles

The P proteins of GII.17 DG42 strains were made as described previously^37^,^38^. A cysteine-containing peptide was linked to the C (P-CDCRGDCFC) terminus of the P domains to enhance P-particle formation. The cDNAs encoding the capsid P domain were cloned into the expression vector pGEX-4T-1 (Amersham Biosciences, Piscataway, NJ) between Sal I and Not I sites. After sequence confirmation, the P proteins were expressed in E. coli. Briefly, the BL21 cultures were induced by IPTG (isopropyl-β-D-thiogalactopyranoside) (0.4 mM) at room temperature (22 °C) overnight. The recombinant P domain-GST fusion proteins were purified using Glutathione Sepharose 4 Fast Flow resin (7 Sea Pharmatech Co., Ltd, CN) according to the manufacturer’s instructions. GST was removed from the P proteins by thrombin (GE Healthcare life Sciences, NJ, USA) cleavage on beads at room temperature overnight. The P-particle formation was confirmed by gel filtration, using a Superdex 200 (GE Healthcare Life-Sciences, Piscataway, NJ) size exclusion column, during which the P particles formed a peak at ~830 kDa.

### Construction of the GII.17 P domain homology model

A GII.17 P domain 3 dimensional model was constructed by the homology modeling database SWISS-MODEL (http://swissmodel.expasy.org/) using the crystal structure of GII.10 P domain (PDB code: 3ONU) as the template. The model was used to show the binding pocket and locations of the mutations of the GII.17 variant DG42 compared with the CS-E1 (2002) and the Kawasaki 323 (2014) variants are shown.

### Detection of Histo-Blood Group Antigens in Saliva

The HBGA phenotypes of A, B, H, Le^a^, Le^b^, Le^x^, and Le^y^ antigens of the saliva samples were determined by EIA assays using the corresponding monoclonal antibodies against individual HBGAs, as described previously^39^. Briefly, boiled saliva (1:1000) was coated on high binding ELISA plates (Costar, Corning, NY, US). After blocking with 5% nonfat milk-PBS, 100μl diluted (1:300) monoclonal antibodies specific for A (Z2A), B (Z5H-2), H (87-N) (Santa Cruz, CA), Le^a^ (BG-5), Le^b^ (BG-6), Le^x^ (BG-7) and Le^y^ (BG-8) antigens (Signet Laboratories Inc., Dedham, MA) were added. Then HRP conjugated goat anti-mice IgG or IgM (1:3000) (Boster biological Techology, Pleasanton, CA) were added. The signal intensities were displayed by adding HRP substrate reagents for 10 min (Tiangen biotech co. ltd, Beijing, CN), and stopped by addition of 2M H_3_PO_4_. The cut-off of a positive signal was OD_450_ = 0.1. Well-characterized positive and negative samples were added in each plate as quality control.

### HBGA binding assay

Saliva-based P particle-HBGA binding assay was performed. Boiled saliva samples were diluted 1:1000 with 1XPBS and coated onto 96-well microtiter plates at 4 °C overnight. After blocking with 5% nonfat dry milk, P particles of GII.17 variant DG42 were added and incubated at 37 °C for 1 hr. The bound P particles were detected using guinea pig anti-NoV (1:2000) sera^39^, followed by HRP-conjugated goat anti-guinea pig IgG (AB clonal Biotechnology Co., Ltd, CN). The signals were developed using a TMB substrate kit (Beyotime Biotechnology Co., Ltd, Shanghai, CN).

### Statistical Analysis

Categorical data were compared by using the Fisher exact test with 2-tailed significance. Unadjusted odds ratios (OR) and 95% confidence intervals (CIs) were calculated using SPSS 20.0 for Windows 7 (SPSS Inc., Chicago, IL, USA).

## Additional Information

**How to cite this article**: Zhang, X.-F. *et al*. An outbreak caused by GII.17 norovirus with a wide spectrum of HBGA-associated susceptibility. *Sci. Rep.* **5**, 17687; doi: 10.1038/srep17687 (2015).

## Figures and Tables

**Table 1:** Stool Specimen Testing Summary.

**Subject**	**Sample**	**Collection date**	Norovirus PCR*	**Norovirus genotyping**§
Student 1	Vomitus	2014/12/22	GII, +	GII-17
Student 2	Stool	2014/12/22	GII, +	GII-17
Student 3	Stool	2014/12/22	GII, +	GII-17
Student 4	Stool	2014/12/22	GII, +	GII-17
Student 5	Stool	2014/12/22	GII, +	GII-17
Student 6	Stool	2014/12/22	GII, +	GII-17
Student 7	Stool	2014/12/22	GII, +	GII-17
Student 8	Stool	2014/12/22	GII, +	GII-17
Student 9	Stool	2014/12/22	GII, +	GII-17
Student 10	Stool	2014/12/22	GII, +	GII-17
Student 11	Stool	2014/12/22	GII, +	GII-17
Student 12	Stool	2014/12/22	GII, +	NP
Student 13	Vomitus	2014/12/22	—	NP
Student 14	Vomitus	2014/12/22	—	NP
Student 15	Vomitus	2014/12/22	—	NP
Student 16	Stool	2014/12/22	—	NP
Student 17	Stool	2014/12/22	—	NP
Student 18	Stool	2014/12/22	—	NP
Teacher 1	Stool	2014/12/22	GII, +	GII-17
Food worker 1†	Stool	2014/12/22	GII, +	GII-17
Food worker 2	Stool	2014/12/22	—	NP

^*^performed by CDC of Dongguan city, Guangdong Province;

^§^performed by CDC of Guangdong Province; NP = not performed;

^†^8–10 episodes diarrhea per day since 3 days before outbreak.

**Table 2:** Distribution of ABO bloodtype, lewis and secretor status among 192 participants in a case–control study of a norovirus gastroenteritis outbreak in China, 2014.

**Status**	**No. (%) of cases**	**No. (%) of controls**	**Total no. (%)**	**χ**^**2**^ **value**	**P value**
**Blood type**†	**n = 67**	**n = 102**	**n = 169**	3.337	0.340
A	23 (34.3)	23 (22.5)	46 (27.2)		
B	18 (26.9)	29 (28.4)	47 (27.8)		
O	23 (34.3)	46 (45.1)	69 (40.8)		
AB	3 (4.5)	4 (3.9)	7 (4.1)		
**Lewis status**	**n = 69**	**n = 123**	**n = 192**	8.725	0.026*
Le^b+^/Le^y+^	64 (92.8)	96 (78.0)	160 (83.3)		
Le^a+^/Le^x+^	2 (2.9)	16 (13.0)	18 (9.4)		
Le^a+b+^/Le^x+y+^	3 (4.3)	6 (4.9)	9 (4.8)		
Le^a-b-x-y-^	0 (0)	5 (4.1)	5 (2.6)		
**Secretor status**	**n = 69**	**n = 123**	**n = 192**	8.42	0.003**
Secretor	67 (97.1)	102 (82.9)	169 (88.0)		
Nonsecretor	2 (2.9)	21 (17.1)	23 (12.0)		

Fisher exact test, *P<0.05, **P<0.01.

^†^Blood types were only determined for secretor-positive individuals.

**Table 3:** Influence of ABO blood type, lewis and secretor status on risk for norovirus GII.17 symptomatic infection.

**Status**	**OR (95% CI)**	**P value**
Blood type†, n = 169		
A, n = 46	1.80 (0.91–3.56)	0.11
B, n = 47	0.93 (0.46–1.85)	0.86
O, n = 69	0.64 (0.34–1.20)	0.20
AB, n = 7	1.15 (0.25–5.30)	1.00
Secretor status, n = 192		
Secretor, n = 169	6.90 (1.57–30.38)	0.003**
Nonsecretor, n = 23	0.15 (0.03–0.64)	0.003**
Lewis, n = 192		
Le^a-b+^/Le^x-y+^, n = 160	3.60 (1.32–9.84)	0.01*
Le^a+b-^/Le^x+y-^, n = 18	0.20 (0.04–0.90)	0.02*
Le^a+b+^/Le^x+y+^, n = 9	0.89 (0.22–3.66)	1.00
Le^a-b-x-y-^, n = 5	Not applicable	0.16

^†^Blood types were only determined for secretor-positive individuals.

Unadjusted odds ratios (OR) and 95% confidence intervals (CIs),*P < 0.05, **P < 0.01.

**Table 4:** Relationship between clinical symptoms of norovirus infection and secretor status and blood type distribution among 69 participants in GII.17 norovirus gastroenteritis outbreak.

**Data**	**No. (%) persons reporting symptom**					
	**Headache**	**Nausea**	**Vomiting**	**abdominal distension**	**abdominal pain**	**Diarrhea**
Blood-type†						
A, n = 23	2 (8.7)	13 (56.5)	21 (91.3)	8 (34.8)	22 (95.7)	1 (4.3)
B, n = 18	3 (16.7)	10 (55.6)	18 (100.0)	5 (27.8)	15 (83.3)	2 (11.1)
O, n = 23	2 (8.7)	15 (65.2)	22 (95.6)	6 (26.1)	20 (87.0)	0 (0.0)
AB, n = 3	0 (0.0)	0 (0.0)	3 (100.0)	0 (0.0)	1 (33.3)	0 (0.0)
Secretor, n = 67	7 (10)	38 (56.7)	64 (95.5)	19 (28.4)	58 (86.6)	3 (4.5)
Nonsecretor, n = 2	0 (0)	1 (50.0)	2 (100)	0 (0.0)	2 (100.0)	0 (0.0)

^†^Blood types were only determined for secretor-positive individuals.
